# Acute myasthenia caused by lithium: a case report

**DOI:** 10.3389/fpsyt.2025.1467267

**Published:** 2025-04-09

**Authors:** Jiashu Yao, Ruihuan Ye, Xihao Mao, Mingmin Jin, Rong Yan, Yuedi Shen, Ning Dai, Wei Chen

**Affiliations:** ^1^ Department of Psychiatry, Sir Run Run Shaw Hospital, School of Medicine, Zhejiang University, Hangzhou, Zhejiang, China; ^2^ Department of Psychiatry, Shaoxing 7th People’s Hospital, Shaoxing, Zhejiang, China; ^3^ The Affiliated Hospital of Hangzhou Normal University, Hangzhou Normal University, Hangzhou, Zhejiang, China; ^4^ Department of Gastroenterology, Sir Run Run Shaw Hospital, School of Medicine, Zhejiang University, Hangzhou, Zhejiang, China

**Keywords:** lithium, myasthenia, side effect, synaptic function, neuromuscular junction

## Abstract

We report the case of an 18-year-old female patient with bipolar disorder who developed recurrent acute myasthenia after taking lithium. The myasthenia resolved upon discontinuation of lithium, and this occurred twice. The results of the cranial magnetic resonance imaging (MRI), multiple sleep latency test, polysomnography, ambulatory electroencephalogram (EEG), and other tests were all negative. In summary, the patient’s acute myasthenia was attributed to lithium use. Unlike previously reported cases of lithium-induced chronic and mild myasthenia symptoms, this case involved acute and widespread myasthenia with falls as the primary manifestation. The mechanism may be related to the effect of lithium on acetylcholine receptor (AChR) metabolism, neurotransmitter release, and neuromuscular junction (NMJ) stability.

## Introduction

Lithium is a commonly used to treat bipolar disorder with tremor being the most frequent neuromuscular side effect (1). Lithium intoxication can lead to symptoms such as sluggishness, ataxia, confusion, agitation, and neuromuscular excitability, manifesting as coarse tremors, fasciculations, or myoclonic jerks (2). However, lithium-induced myasthenia is relatively rare, with only six cases reported to date (3–8). These cases primarily involved chronic symptoms, such as exertional weakness, ptosis, diplopia, slurred speech, and dysphagia. In contrast, we report a case of lithium-induced acute myasthenia with recurrent falls as the primary manifestation.

### History of present illness

An 18-year-old female patient with a diagnosis of bipolar disorder was admitted to the psychiatric ward after experiencing recurrent mood swings for five years. Six months prior, she had a recurrence of depression, characterized by a lack of motivation, poor sleep, and suicidal thoughts. After starting lithium carbonate (0.3 g once daily), fluoxetine (40 mg once daily), aripiprazole (10 mg nightly), and zopiclone (7.5 mg before bed), her mood improved, motivation increased, and suicidal thoughts diminished. However, she began experiencing recurrent falls every few days.

The falls were preceded by sudden generalized weakness, with the patient falling to the ground for approximately one minute. During these episodes, her speech became very soft, and she was unable to lift her head. There were no obvious triggers, no visual disturbances, no loss of consciousness, no chest pain, palpitations, ptosis, or diplopia, no urinary or fecal incontinence. She was able to recall the events in detail.

Two months ago, her lithium dose was increased to 0.3 g three times a day, lamotrigine (25 mg once daily) was added, while zopiclone 7.5 mg before bed. Although her mood stabilized, with a significant reduction in depressive symptoms and disappearance of suicidal thoughts, the frequency of fall increased from once every few days to several times a day. Each episode of weakness lasted from 1 minute to half a day. She typically fell on her knees, causing bruising, but no other visible injuries were noted. A Holter dynamic electrocardiogram revealed a sinus heart rate with bradycardia accounting for 36.5% of the time, sinus tachycardia for 11.6%, and a longest R-R interval of 1.5 seconds. Chest CT, head CT, 24-hour EEG, and 24-hour blood pressure monitoring were all normal.

After discontinuing lithium, the patient stopped experiencing falls, though other medications remained unchanged. However, her depressive symptoms and suicidal thoughts resurfaced. Consequently, One month ago, lurasidone (20mg once nightly) was added, and lamotrigine was increased to 50mg daily. One week later, the medication was further adjusted: lamotrigine (75mg twice daily), lurasidone (40mg nightly), tandospirone (10mg three times daily), and zolpidem (10mg once before bed). Her mood improved, and she became more positive.

However, her sleepiness worsened following the medication adjustment, and the lurasidone was considered the culprit. It was discontinued two weeks ago, and lamotrigine was increased to 100mg twice daily. Her Sleepiness improved, but her depression worsened, and mood swings became more pronounced.

### Treatment and follow-up in the hospital

During this hospitalization, lithium carbonate (0.3 g twice daily) was reintroduced to control the symptoms of depression, and her mood became somewhat more stable. However, her falls recurred, occurring one to several times a day. As with previous episodes, her speech volume was low, and she was unable to lift her head. There were no episodes of amaurosis, loss of consciousness, chest tightness, chest pain, or palpitation, no ptosis, or diplopia, and no urinary or fecal incontinence. Neurological examination revealed that her muscle strength in the extremities was graded at 3-, tendon reflexes were ++, and no Babinski signs were elicited bilaterally.

Relevant examinations included cranial MRI, which showed small ischemic foci in bilateral frontal lobes; cardiac ultrasound which revealed mild mitral and tricuspid regurgitation; and a Holter dynamic electrocardiogram, which revealed a sinus heart rate with bradycardia accounting for 63.3% of the time (heart rate 40-141 beats/min, average 61 beats/min), sinus tachycardia for 4.5% of the total time, and a longest R-R interval of 1.5 seconds. A 24-hour dynamic EEG was normal, and blood routine, biochemistry, vitamin B12, folate, and thyroid function were normal; A prone-standing blood pressure test was negative. Multiple nap tests revealed an average sleep latency of 19.3 min, with no Sleep Onset Rapid Eye Movement Period (SOREMP). Polysomnography from the previous night was also negative.

After the discussing the case with neurologists and cardiologists, lithium was considered the likely cause of the patient’s fall. After discontinuing lithium carbonate, the fall ceased.

The patient’s therapeutic regimen was adjusted to lamotrigine (100 mg twice daily), followed by oxcarbazepine (0.45 g twice daily). Her mood significantly stabilized and remained stable throughout the 1.5 years of follow-up, with no recurrence of falls or weakness.

### Medical history

None.

### Personal history

Both of her parents had short tempers. When she was a child, her father was often absent, and her mother would beat her. She currently has a poor relationship with her parents and is prone to conflict with them. She is a perfectionist and becomes upset if things do not go as planned. She is graduating from high school and will be attending college soon. Under great academic pressure, her performance has declined, and she feels that teachers and classmates look down on her, leading to low self-esteem.

## Discussion

In this case, we hypothesized that the patient’s recurrent falls were caused by the lithium for two primary reasons.

**Figure 1:**
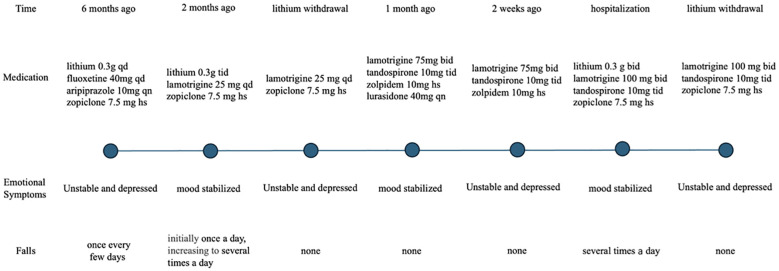
Timeline on medications, emotional symptoms and falls.

The first and most compelling reason is the temporal relationship between the administration of lithium and the onset of the falls. The patient’s falls began after starting lithium and ceased upon discontinuation of the drug, which occurred twice (**Figure 1**). At the time of the first fall, the medications administered included lithium, fluoxetine, aripiprazole, lamotrigine, and zopiclone. Among these, only lithium and aripiprazole have been associated with an increased risk of myasthenia, while the others have not (9). Medication changes before the first fall included the discontinuation of aripiprazole and fluoxetine, and an increase in the lithium dose. At the time of the second fall, only lithium was increased, and the falls ceased immediately upon lithium discontinuation, with no other medication changes.

The second reason supporting our hypothesis is that no symptoms or examinations indicated that the falls were caused by another condition. The patient denied experiencing chest tightness, palpitations, or visual disturbances before the falls, and there was no loss of consciousness at the time of the falls. Cardiac evaluations, including Holter dynamic ECG, cardiac ultrasound, and blood pressure measurements (both prone and ambulatory), were all negative, ruling out cardiac syncope due to arrhythmia, valvular heart disease, or orthostatic hypotension. Additionally, the patient was able to recall the details of each fall and did not experience a loss of consciousness during the episodes and two ambulatory EEGs normal, excluding seizures as a potential cause. The patient did not report daytime sleepiness or sleep-related episodes, and the results of multiple sleep latency tests and polysomnography were negative, ruling out narcolepsy. Furthermore, there was no history of thyroid disease, and her current thyroid function tests were normal, making hypothyroidism unlikely. Conversion disorder was also not considered, as the patient showed no psychological triggers or disturbances in the integration of motor, sensory, or cognitive functions prior to the falls.

To date, six cases of lithium-induced myasthenic syndrome have been reported. The first five cases (3–7)were all characterized by chronic symptoms such as exertional weakness, ptosis, diplopia, slurred speech, and dysphagia, which were significantly improved or resolved after lithium was discontinued or its dose was reduced. The sixth case, similar to ours, involved acute myasthenia with sudden falls, but the patient had true myasthenia gravis, with lithium acting only as a precipitating factor (8). While the first five cases of lithium-induced myasthenia primarily presented with chronic symptoms and more localized muscle involvement, particularly affecting the eye and bulbar muscles, our case differed in that the patient experienced multiple episodes of acute weakness involving widespread muscle groups, including the limbs, neck, and bulbar muscles. Each episode was characterized by an abrupt onset of generalized weakness and falls. The heterogeneity in the clinical presentation of lithium-induced myasthenia is evident from these cases.

Lithium-induced myasthenia may involve several mechanisms that affect neuromuscular transmission and postsynaptic stability. First, lithium can reduce the number of acetylcholine receptors (AChRs) in skeletal muscle, potentially by affecting their synthesis, degradation, or transport (10). Lithium can also interfere with AChR metabolism, decreasing receptor stability and impairing synaptic transmission (11). Additionally, lithium inhibits agrin-induced AChR aggregation by disrupting a late step in the agrin signaling pathway, leading to abnormal AChR distribution and impaired synaptic transmission, which may contribute to muscle weakness (12). Lithium may also interfere with acetylcholine synthesis and release from presynaptic terminals, reducing neurotransmitter availability and further impairing signal transmission (13). Furthermore, lithium enhances the effects of neuromuscular blocking agents, such as succinylcholine and pancuronium, which potentiate synaptic inhibition and weaken muscle strength (14, 15). Finally, lithium appears to have a more pronounced effect on neuromuscular junctions (NMJs) in denervated muscles, exacerbating the disruption of synaptic stability and worsening muscle weakness, particularly under pathological conditions (16). These combined effects on AChR metabolism, neurotransmitter release, and NMJ stability likely contribute to the development of lithium-induced myasthenia.

The limitation of this case is that repetitive nerve stimulation or single-fiber electromyography was not performed during the patient’s episodes of muscle weakness to confirm the presence of myasthenia. Additionally, despite thorough differential diagnoses, the possibility of other medications, diseases, or psychological factors contributing to myasthenia gravis cannot be completely excluded.

This case highlights the need for increased awareness among psychiatrists regarding the possibility of lithium-induced myasthenia, a potentially overlooked side effect that requires careful monitoring in patients prescribed lithium. The exact mechanisms by which lithium induces myasthenia are still not fully understood and warrant further investigation in future studies.

## Data Availability

The raw data supporting the conclusions of this article will be made available by the authors, without undue reservation.
